# Longitudinal trends of and factors associated with inappropriate antibiotic prescribing for non-bacterial acute respiratory tract infection in Japan: A retrospective claims database study, 2012–2017

**DOI:** 10.1371/journal.pone.0223835

**Published:** 2019-10-16

**Authors:** Yuki Kimura, Haruhisa Fukuda, Kayoko Hayakawa, Satoshi Ide, Masayuki Ota, Sho Saito, Masahiro Ishikane, Yoshiki Kusama, Nobuaki Matsunaga, Norio Ohmagari

**Affiliations:** 1 AMR Clinical Reference Center, National Center for Global Health and Medicine, Tokyo, Japan; 2 Department of Health Care Administration and Management, Kyushu University Graduate School of Medical Sciences, Fukuoka, Japan; 3 Disease Control and Prevention Center, National Center for Global Health and Medicine, Tokyo, Japan; University of Campania, ITALY

## Abstract

**Background:**

Inappropriate antibiotic prescribing is a cause of antimicrobial resistance. Acute Respiratory Tract Infections (ARTI) are common diseases for those antibiotics are most likely prescribed in outpatient setting.

**Objectives:**

To clarify factors associated with antibiotic prescribing for non-bacterial acute respiratory tract infections (NB-ARTI) and identify targets for reducing inappropriate prescribing for NB-ARTI in Japan.

**Methods:**

We conducted a retrospective, observational study using longitudinal claims data between April 2012 and June 2017. We assessed the rate of and factors associated with inappropriate antibiotic prescribing in outpatient settings for all NB-ARTI consultations included in the database.

**Results:**

The mean monthly antibiotic prescribing rate per 100 NB-ARTI consultations during the study period was 31.65. The monthly antibiotic prescribing rate per 100 NB-ARTI consultations decreased by 19.2% from April 2012 to June 2017. Adolescents (13–18 years) and adults of working age (19–29 and 30–39 years) were more likely prescribed antibiotics compared with elderly patients ≥ 60 years (aOR: 1.493 [95%CI: 1.482–1.503], 1.585 [95%CI: 1.575–1.595], and 1.507 [95%CI: 1.498–1.516], respectively). Outpatient clinics registered as internal medicine or ear, nose, and throat specialty were more likely to prescribe antibiotics than those of paediatric specialty or other specialties. Among health facility type, clinics without beds (aOR 2.123 [95%CI: 2.113–2.133]) and clinics with beds (aOR: 1.752 [95%CI: 1.7371–1.767]) prescribed significantly more antibiotics for NB-ARTI than outpatient departments inside general hospitals.

**Conclusions:**

Inappropriate antibiotic prescribing for NB-ARTI is common in Japan. Although the antibiotic prescribing rate has decreased, further interventions are required to promote antimicrobial stewardship (ASP). Education and awareness for adults and promotion of ASP among physicians in clinics without beds are key drivers to reduce inappropriate antibiotic prescribing in Japan.

## Introduction

Inappropriate use of antimicrobials is one of the drivers of the emergence of antimicrobial resistance (AMR). The Japanese government published the National Action Plan on AMR (NAP) in 2016, in which promoting appropriate antimicrobial use is listed as one of the main strategies [1]. Surveillance data of antibiotic consumption (AMU) in Japan showed a rate of 14.19 DDDs/1,000 inhabitants/day in 2015, which was not as high as other countries in Europe [2]. However, oral AMU accounted for 92.6% of total AMU; 77.1% of oral AMU consisted of broad-spectrum antibiotics such as third-generation cephalosporins, macrolides, and fluoroquinolones in Japan [3], suggesting that oral AMU is an important target of antimicrobial stewardship (ASP).

Acute respiratory tract infections (ARTI) are common diseases in primary care and many patients with ARTI symptoms are prescribed antibiotics despite the fact that the majority of these infections are caused by viruses. In the US, approximately 30% of antibiotic prescriptions in emergency departments and physicians’ office was reported to be unnecessary and 50% of antibiotic prescriptions for ARTI was deemed to be inappropriate [4]. It was also reported that antibiotics were prescribed inappropriately for 25% and 63% of viral upper respiratory tract infections and bronchitis, respectively, during influenza seasons in the US [5]. Another study conducted in England reported an inappropriate antibiotic prescribing rate of 44.5% of ARTI consultations at general practices in 2016–2017 [6]. In Italy, 66.5% of the antibiotic prescriptions given to adult patients were not indicated by the guidelines for the treatment of respiratory tract infections [7]. In a study in Norway on antibiotic prescribing for acute respiratory tract infections in primary care out-of-hours service, 2310 (34.2%) of 6757 cases of ARTIs resulted in an antibiotic prescription [8].

Several studies on antibiotic prescribing practice for ARTI in Japan have been reported thus far. A previous study using local claims data from 2005 showed that antibiotics were prescribed in 60% of ARTI consultations in the outpatient setting [9]. Yoshida et al. reported that 66.4% of children younger than 6 years who visited health facilities were prescribed antibiotics for ARTI conditions using the retrospective claims database. They suggested that antibiotic prescriptions for non-bacterial acute upper respiratory infections (NB-ARTI) were associated with male sex, non-paediatrics, out-of-hour visit, and clinic as health facility type compared with hospitals [10]. Recently, a study using data from a claims database from 2013 to 2015 reported that 52.7% of ARTI patients received antibiotics [11]. These studies have provided information that inappropriate antibiotic prescribing for ARTI is also common in Japan. However, longitudinal information on antimicrobial prescription patterns focusing on both children and adults nationwide is lacking. Moreover, factors associated with inappropriate antibiotic prescribing for NB-ARTI in outpatient settings have not been well studied. Therefore, this study aimed to identify longitudinal trends in antibiotic prescribing in NB-ARTI patients and clarify factors associated with inappropriate antibiotic prescribing for NB-ARTI.

## Materials and methods

### Data source

This is a retrospective observational study using administrative claims data from April 2012 to June 2017 obtained from JMDC Inc. (Tokyo, Japan). The JMDC database contains anonymous claims data of 5.8 million (as of June 2018) corporate employees covered by the employees’ health insurance plans and their family members aged younger than 75 years, that roughly represents 5% of Japanese population. Data consist of patient characteristics (sex, year and month of birth, and insured status), consultation information (date of visit, time zone of visit, initial date of a diagnosis given, and diagnosis in the ICD-10 code and Japanese Insurance Code), medication information (name of prescribed medicine, date of prescription, and name and date of medical procedure given), and facility information (number of beds, specialty of facility, and facility operation type).

### Assessment of appropriate antibiotic prescribing

To estimate appropriate antibiotic prescribing, antibiotic appropriateness for every possible diagnosis of the Japanese National Health Insurance (NHI) was assessed by a panel of six infectious disease specialists at the Disease Control and Prevention Center, National Center for Global Health and Medicine Hospital, Tokyo, Japan. Japanese NHI code is more detailed than ICD 10, and each ICD-10 code include equal or more than one NHI code. Each diagnosis of the NHI was assigned to two experts who independently assessed whether antibiotic treatment for a diagnosis was necessary. If the assessment for each diagnosis between the two experts did not match, then all six experts discussed at a round table and made a collective decision on the appropriateness of these diagnoses (S1 Table). The validity of this approach has been demonstrated in previous studies [4,12].

### Data processing

To calculate the antibiotic prescribing rate at NB-ARTI consultations, we extracted the dataset comprising ARTI consultations from the original database. These data were associated with visits to outpatient clinics or other outpatient departments of hospitals by patients diagnosed with ARTI (ICD-10; J00-06, J20-22)[13]. We then excluded consultations at which any diagnosis was made that required antibiotic treatment, as defined above. In the present study we did not consider the patients’ history of chronic bronchitis and COPD, because of limitations necessitated by the large size of the dataset used in the study. In the NHI claims system, the date of diagnosis is provided separately from the consultation date and diagnoses usually remain in the claims database unless the health facilities delete them. To prevent overestimation of the inappropriate antibiotic prescribing rate, we only included diagnoses made by 30 days prior to the consultation date, on the basis of a sensitivity analysis of the antibiotic prescribing rate according to changes in linkage of the diagnosis and consultation datasets. Because of the difficulty in identifying follow-up visits to ARTI consultations in the NHI claims system, we did not exclude these. A dataset for the prescribed antibiotics was prepared separately to the NB-ARTI consultation dataset. We included oral systemic antibiotics of the J01 class of the WHO’s Anatomical Therapeutic Chemical (ATC) Classification System in the analysis. According to the NAP, a 50% reduction in the use of oral third-generation cephalosporins (ATC: J01DD), macrolides (ATC: J01FA), and fluoroquinolones (ATC: J01MA) is listed as one outcome index, and we categorized these three oral antibiotics as broad-spectrum antibiotics in this study. We linked the antibiotic prescription dataset to the NB-ARTI consultation dataset if an antibiotic was prescribed on the same date as the NB-ARTI consultation. Because NB-ARTI diagnosis dates were quite vague, we used the consultation dates to link the prescription dataset. Unfortunately, in Japan, point-of-care tests are not commonly utilized in outpatient settings and clinicians usually prescribe antibiotics without confirmation of a bacterial infection. Therefore, we did not incorporate an analysis of the link between clinical laboratory data and antibiotic prescribing in the present study. We then analysed the linked dataset to determine whether the antibiotic had been prescribed for NB-ARTI.

### Statistical analysis

We analysed the monthly antibiotic prescribing rates per 100 NB-ARTI consultations conducted in outpatient clinics. In this analysis, the numerator was the number of consultations during which an antibiotic was prescribed for NB-ARTI at a consultation during each month, and the denominator was the number of consultations at which NB-ARTI was diagnosed during the same month. We then conducted a simple regression analysis to compare the NB-ARTI diagnosis rate per ARTI consultation and the antibiotic prescribing rate per NB-ARTI consultation, to determine whether the reduction in antibiotic prescribing rate was affected by the reduction in the number of NB-ARTI diagnoses. Another simple regression analysis was carried out to compare the longitudinal trends in antimicrobial prescribing in the various age groups. In bivariate analyse, we compared the antibiotic prescribing rates by patient age group, the pharmacological classification of the antibiotic, and diagnosis. We categorized health facilities into clinics without beds, clinics with up to 19 beds, general hospitals with more than 20 beds, university hospitals, and national or municipal hospitals. Consultations were classified according to whether they were conducted during normal working ours (defined as between 8:00 h and 18:00 h on a weekday) or out-of-hours (between 18:00 h and 8:00 h on weekdays and all day during weekends or holidays). In Japan, NHI covers almost all the population, but in addition, local governments subsidize children’s healthcare, and the policy is usually implemented from the beginning of the fiscal year (April to March). During the study period, almost all the relevant local governments introduced and continued these subsidies. To consider the potential difference in antibiotic prescribing rates due to health subsidies by local governments, patients were categorised into nine age groups: 0–3 years, 4–6 years, 7–12 years, 13–18 years, 19–29 years, 30–39 years, 40–49 years, 50–59 years, and ≥ 60 years. Longitudinal trend of antibiotic prescribing rate was evaluated by the simple linear regression model to analyse the correlation between antibiotic prescribing and time change. Multivariate logistic regression analyses were conducted to determine the association between characteristics of patients and health facilities and antibiotic prescribing. All statistical analyses were performed using Stata 15.1 (StataCorp, College Station, TX, USA). P values < 0.05 were considered statistically significant.

### Ethics statement

The study was approved by the Institutional Review Board of the National Center for Global Health and Medicine Hospital (approval number NCGM-G-002425-00). The data obtained from JMDC Inc. was completely anonymized before we analyze it. Therefore, subjects’ informed consent was not required.

## Results

### Characteristic of patients and health facilities included in the study

Table 1 summarizes the characteristic of patients and health facilities included in the study. Between April 2012 and June 2017, 26,648,301 ARTI consultations were identified among 8,983,098 outpatients. Of these, 17,208,787 NB-ARTI consultations were conducted at 68,788 health facilities for 3,848,205 outpatients within the age range of 0–75 years (mean 20.0 years, standard deviation 20.138 years). Of the age groups studied, children aged 0–3 years were seen most often, followed by adults aged 30–39 and 40–49 years and elderly aged > 60 years. The majority (88.3%) of NB-ARTI consultations were conducted in clinics without beds.

**Table 1:** Characteristics of patients and healthcare facilities included in the study.

			Number of NB-ARTI consultations (N = 17,208,787)	%
**Patient Characteristics**				
	Male		8,987,136	52.2
	Mean age at consultation		20.0	-
	Age group			
		0–3	4,974,435	28.9
		4–6	2,270,582	13.2
		7–12	1,983,064	11.5
		13–18	955,853	5.6
		19–29	1,308,577	7.6
		30–39	1,915,203	11.1
		40–49	1,803,954	10.5
		50–59	1,282,880	7.5
		60-	714,239	4.2
	Insurance status			
		Insured member	4,632,919	26.9
		Family member	12,575,868	73.1
**Facility Characteristics**				
	Specialty			
		Internal medicine	9,148,220	53.2
		Paediatric	5,188,970	30.2
		ENT	1,655,730	9.6
		Others	1,215,867	7.1
	Operation type			
		Clinic without bed	15,194,276	88.3
		Clinic with bed	359,993	2.1
		University hospital	76,036	0.4
		Municipal hospital	315,043	1.8
		Other hospital	1,263,439	7.3
**Diagnosis**				
	Acute bronchitis (J20)		5163280	30.0
	Acute upper respiratory infections (J06)		4981832	29.0
	Acute pharyngitis (J02)		2419782	14.1
	Acute nasopharyngitis (J00)		1028080	6.0
	J06 & J20		847664	4.9
	J02 & J20		584844	3.4
	Acute tonsillitis (J03)		472562	2.8
	J02 & J06		302656	1.8
	J00 & J20		217127	1.3
	J00 & J06		201049	1.2
	others		989911	5.8
**Fiscal year**				
	2012		2,279,159	13.2
	2013		3,330,801	19.4
	2014		3,547,177	20.6
	2015		3,643,026	21.2
	2016		3,647,077	21.2
	2017		761,547	4.4

### Overall antibiotic prescribing rate for NB-ARTI and differences among age groups and diagnoses

**Fig 1:**
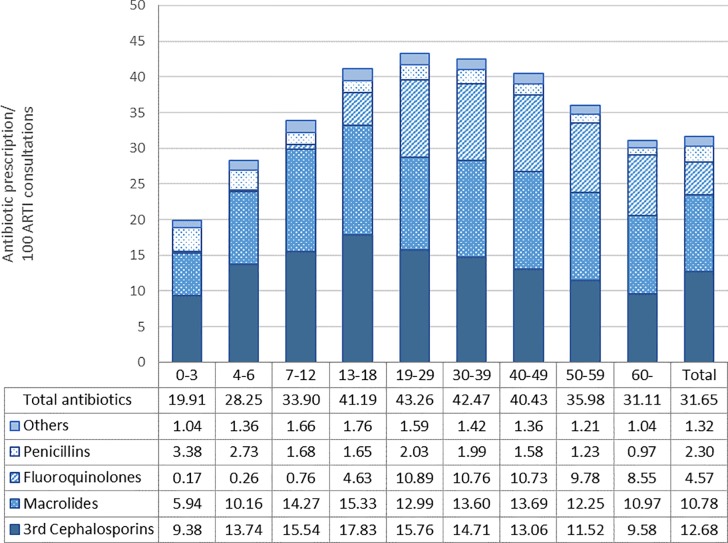
Mean monthly antibiotic prescribing rate for non-bacterial acute respiratory tract infections by age group during the study period.

The mean monthly antibiotic prescribing rate per 100 NB-ARTI consultations during the study period was 31.65 (Fig 1). The results of the sensitivity analysis of antibiotic prescribing rate according to changes in the linkage of the diagnosis and consultation datasets are shown in S2 Table. Approximately 89% of antibiotics prescribed for NB-ARTI during the study period were broad-spectrum antibiotics, specifically third-generation cephalosporins (40.1%), macrolides (34.1%), and fluoroquinolones (14.4%). The prescribing rate varied by patient age group; adults aged 19–29 years had the highest rate (43.26 antibiotic prescriptions/100 NB-ARTI consultations), while children aged 0–3 years had the lowest rate (19.91 antibiotic prescriptions/100 NB-ARTI consultations) (p < 0.001). Adolescents and working-age adults [i.e. patients aged between 19 and 49 years (age groups: 19–29, 30–39, and 40–49 years)] more frequently received antibiotics for NB-ARTI than the other age groups. Furthermore, working-age adults and the elderly were significantly more often prescribed broad-spectrum antibiotics for NB-ARTI than children (p < 0.001).

**Fig 2:**
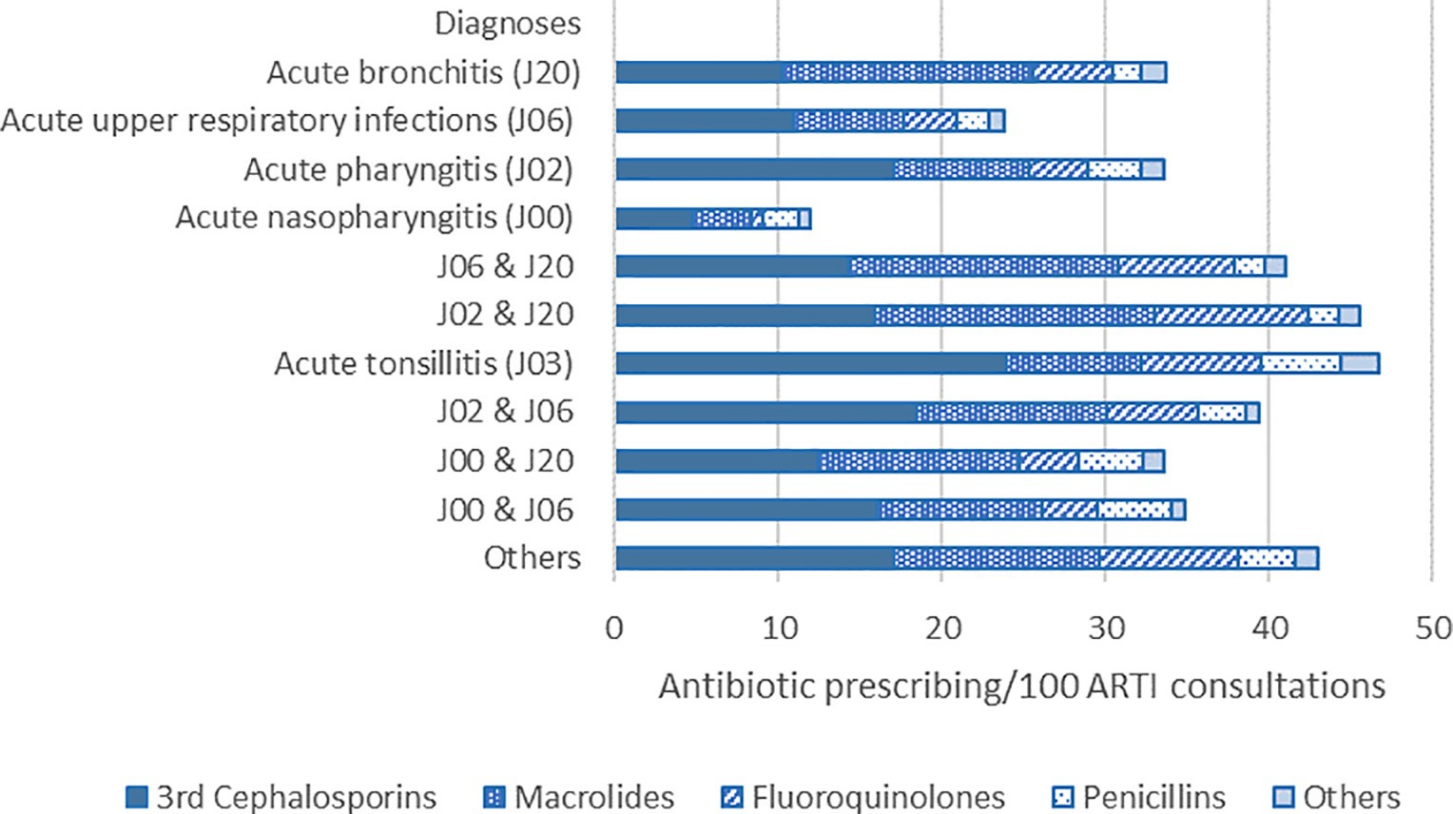
Overall antibiotic prescribing rate for non-bacterial acute respiratory tract infections by diagnosis during the study period.

Commonly reported diagnoses among NB-ARTI consultations were “acute bronchitis” (ICD-10: J20, accounted for 30.0% of NB-ARTI consultations in the study period), “acute upper respiratory infections” (ICD-10: J06, 29.0%), and “acute pharyngitis” (ICD-10: J02, 14.1%) (Fig 2). Antibiotics were most commonly prescribed for “acute tonsillitis” (46.79 antibiotic prescriptions/100 NB-ARTI consultations), and the combination of two ARTI diagnoses (e.g. **“**acute upper respiratory infections” and “acute bronchitis”, “acute pharyngitis”, and “acute bronchitis”) yielded higher antibiotic prescribing rates than consultations with single ARTI diagnoses such as “acute upper respiratory infections” (24.03 antibiotic prescriptions/100 NB-ARTI consultations) or “acute pharyngitis” (12.03 antibiotic prescriptions/100 NB-ARTI consultations).

### Longitudinal trend of antibiotic prescribing rate

**Fig 3:**
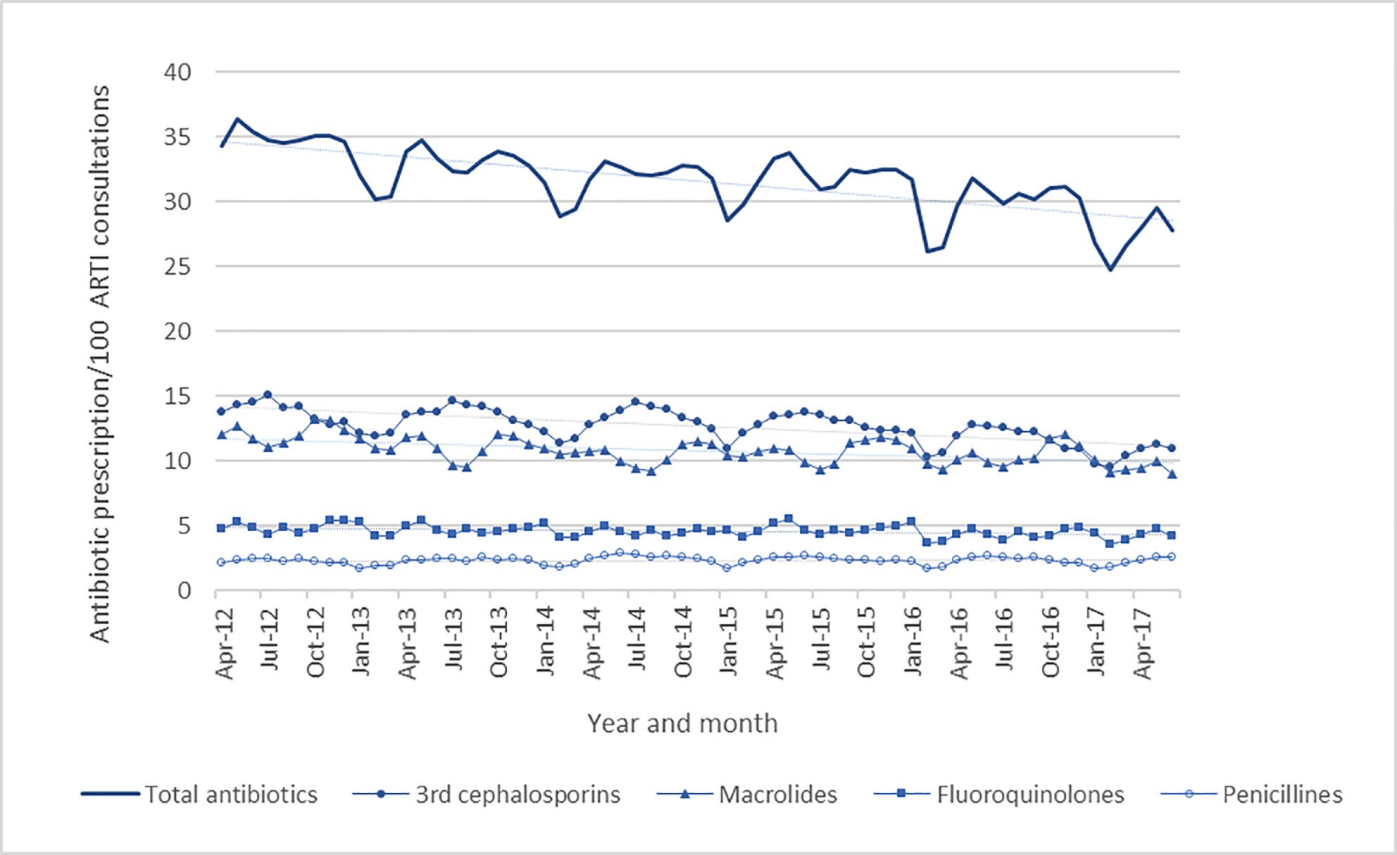
Trend of monthly antibiotic prescribing during the study period.

The monthly antibiotic prescribing rate per 100 NB-ARTI consultations decreased by 19.2% from April 2012 (34.36 antibiotic prescriptions/100 NB-ARTI consolations) to June 2017 (27.77 antibiotic prescriptions/100 NB-ARTI consultations). The prescribing rate of third-generation cephalosporins, macrolides, fluoroquinolones, and other antibiotics decreased by 20.5%, 24.9%, 13.0%, and 32.9%, respectively, between April 2012 and June 2017 (Fig 3). There were significant negative trends in monthly prescribing rates of total antibiotics (Coefficient = −0.09839), third-generation cephalosporins (Coefficient = −0.04857), macrolides (Coefficient = −0.03136), and fluoroquinolones (Coefficient = −0.00964). In contrast, there was no significant trend in the monthly prescribing rate of penicillins, which increased by 18.2% (Coefficient = 0.000831) (Table 2). Simple regression analysis yielded a fixed coefficient of 0.009 and a regression coefficient of −0.234 (p = 0.811); therefore, the negative trend in antibiotic prescribing rate was not associated with the trend of the number of NB-ARTI diagnoses to decrease. When the longitudinal changes in antibiotic prescribing among the various age groups were compared, the regression coefficient tended to be negative for younger age groups (S1 Fig).

**Table 2:** Longitudinal trend of antibiotic prescribing rate between April 2012 and June 2017*.

	Coef.	P value	R-squared
**Total antibiotic prescribing for NB-ARTI**	-0.09839	< 0.001	0.529
**Third-generation cephalosporins (J01DD)**	-0.04857	< 0.001	0.4703
**Macrolides (J01FA)**	-0.03136	< 0.001	0.3150
**Fluoroquinolones (J01MA)**	-0.00964	0.001	0.1597
**Penicillins (J01C)**	0.000831	0.676	0.0029

*Evaluated by the correlation between antibiotic prescribing and time change.

Abbreviations: NB-ARTI, non-bacterial acute respiratory tract infection; Coef, coefficient

### Factors associated with inappropriate antibiotic prescribing for NB-ARTI

As shown in Table 3, multivariate logistic regression analysis indicated that adolescent (13–18 years) and working-age patients (19–29 and 30–39 years) were more likely prescribed antibiotics compared with elderly patients ≥ 60 years (aOR: 1.493 [95%CI: 1.482–1.503], 1.585[95%CI: 1.575–1.595], and 1.507 [95%CI: 1.498–1.516], respectively). Registered members of the NHI were slightly more likely prescribed antibiotics than their dependents (aOR: 1.108 [95%CI:1.104–1.112]. Outpatient clinics registered as internal medicine specialty (aOR: 1.167 [95%CI: 1.162–1.172]) or ear, nose, and throat (ENT) specialty (aOR: 1.424 [95%CI: 1.416–1.432]) were more likely to prescribe antibiotics than those with paediatric specialty or other specialties. Among health facility type, clinics without beds (aOR 2.123 [95%CI: 2.113–2.133]) and clinics with beds (aOR: 1.752 [95%CI: 1.737–1.767]) prescribed significantly more antibiotics for NB-ARTI than outpatient departments inside general hospitals. Outpatient departments inside university hospitals and national and municipal hospitals were less likely to prescribe antibiotics than those in general hospitals. Regarding visiting hours, off-hour consultations were slightly less related to antibiotic prescribing than in-hour consultations (aOR: 0.963 [95%CI: 0.960–0.967]). Diagnosis of “acute tonsillitis” (aOR: 1.460 [95%CI: 1.451–1.469]) and combination of “acute pharyngitis” and “acute bronchitis” (aOR: 1.305 [95%CI: 1.298–1.312]) were more likely associated with antibiotic prescribing compared with diagnosis of “acute bronchitis.” After seasonal fluctuations were taken into consideration, inappropriate antibiotic prescribing showed a tendency to decrease annually.

**Table 3:** Multivariate analysis of predictors of antibiotic prescribing for non-bacterial acute respiratory tract infections.

		Crude OR	(	95% CI			)	Adjusted OR	(	95% CI			)
**Sex**													
	Male	1						1					
	Female	0.954	(	0.952	-	0.956	)	0.957	(	0.955	-	0.960	)
**Age group**													
	0–3	0.547	(	0.544	-	0.550	)	0.644	(	0.640	-	0.648	)
	4–6	0.855	(	0.850	-	0.860	)	0.959	(	0.952	-	0.965	)
	7–12	1.079	(	1.072	-	1.085	)	1.174	(	1.167	-	1.182	)
	13–18	1.443	(	1.434	-	1.453	)	1.493	(	1.482	-	1.503	)
	19–29	1.634	(	1.624	-	1.644	)	1.585	(	1.575	-	1.595	)
	30–39	1.596	(	1.586	-	1.605	)	1.507	(	1.498	-	1.516	)
	40–49	1.462	(	1.454	-	1.471	)	1.385	(	1.377	-	1.393	)
	50–59	1.222	(	1.215	-	1.230	)	1.195	(	1.187	-	1.202	)
	60-	1						1					
**Insurance status**													
	Member	1.715	(	1.711	-	1.719	)	1.108	(	1.104	-	1.112	)
	Member's family	1						1					
**Specialty of facility**													
	Internal Medicine	1.313	(	1.307	-	1.318	)	1.167	(	1.162	-	1.172	)
	Pediatrics	0.724	(	0.721	-	0.728	)	0.878	(	0.874	-	0.883	)
	ENT	1.275	(	1.268	-	1.281	)	1.424	(	1.416	-	1.432	)
	Others	1						1					
**Facility operation type**													
	Clinics without beds	1.870	(	1.862	-	1.878	)	2.123	(	2.113	-	2.133	)
	Clinics with beds	1.647	(	1.633	-	1.661	)	1.752	(	1.737	-	1.767	)
	University hospitals	0.684	(	0.670	-	0.698	)	0.739	(	0.724	-	0.755	)
	Public hospitals	0.684	(	0.677	-	0.692	)	0.793	(	0.784	-	0.801	)
	Other hospitals	1						1					
**Visit hours**													
	In-hour visit	1						1					
	Out-of-hour visit	0.956	(	0.953	-	0.959	)	0.963	(	0.960	-	0.967	)
**Diagnosis**													
	Acute bronchitis (J20)	1						1					
	Acute upper respiratory infections (J06)	0.619	(	0.617	-	0.620	)	0.565	(	0.564	-	0.567	)
	Acute pharyngitis (J02)	0.997	(	0.994	-	1.000	)	0.989	(	0.985	-	0.992	)
	Acute nasopharyngitis (J00)	0.267	(	0.266	-	0.269	)	0.272	(	0.270	-	0.274	)
	J06 & J20	1.369	(	1.363	-	1.376	)	1.152	(	1.147	-	1.158	)
	J02 & J20	1.650	(	1.641	-	1.659	)	1.305	(	1.298	-	1.312	)
	Acute tonsillitis (J03)	1.728	(	1.717	-	1.738	)	1.460	(	1.451	-	1.469	)
	J02 & J06	1.284	(	1.275	-	1.294	)	1.049	(	1.041	-	1.057	)
	J00 & J20	0.997	(	0.988	-	1.007	)	1.019	(	1.009	-	1.028	)
	J00 & J06	1.057	(	1.047	-	1.067	)	0.854	(	0.845	-	0.862	)
	others	1.486	(	1.479	-	1.492	)	1.128	(	1.122	-	1.133	)
**Fiscal year**													
	2012	1						1					
	2013	0.937	(	0.934	-	0.941	)	0.931	(	0.928	-	0.935	)
	2014	0.906	(	0.903	-	0.910	)	0.883	(	0.880	-	0.887	)
	2015	0.879	(	0.876	-	0.882	)	0.838	(	0.834	-	0.841	)
	2016	0.813	(	0.810	-	0.816	)	0.765	(	0.762	-	0.767	)
	2017	0.780	(	0.776	-	0.785	)	0.678	(	0.674	-	0.682	)
**Month**													
	January	1						1					
	February	0.902	(	0.897	-	0.906	)	0.927	(	0.923	-	0.932	)
	March	0.952	(	0.947	-	0.956	)	1.002	(	0.997	-	1.007	)
	April	1.094	(	1.089	-	1.099	)	1.268	(	1.262	-	1.274	)
	May	1.165	(	1.159	-	1.170	)	1.343	(	1.336	-	1.350	)
	June	1.099	(	1.094	-	1.104	)	1.313	(	1.306	-	1.320	)
	July	1.096	(	1.091	-	1.102	)	1.294	(	1.287	-	1.301	)
	August	1.104	(	1.098	-	1.111	)	1.276	(	1.269	-	1.284	)
	September	1.104	(	1.123	-	1.135	)	1.304	(	1.297	-	1.311	)
	October	1.150	(	1.144	-	1.155	)	1.289	(	1.282	-	1.295	)
	November	1.153	(	1.147	-	1.158	)	1.264	(	1.258	-	1.271	)
	December	1.118	(	1.113	-	1.124	)	1.217	(	1.211	-	1.223	)

Abbreviations: OR, odds ratio; CI, confidence interval

We conducted further analysis focusing on the prescription pattern by facility specialty. The trend of odds ratio for antibiotic prescription stratified by facility specialty indicated that antibiotic prescribing for NB-ARTI decreased in every specialty. The reduction of antibiotic prescribing among paediatric clinics was substantial; paediatric clinics prescribed only one-third of antibiotics in FY2017 compared with that in FY2012 (Table 4).

**Table 4:** Trend of adjusted odds ratios for antibiotic prescribing by specialty of facility and consultation year*.

	Overall	Internal Medicine	Pediatrics	ENT	Others
**2012**	**1**	**1**	**1**	**1**	**1**
**2013**	**0.931**	**0.932**	**0.918**	**0.982**	**0.923**
**2014**	**0.883**	**0.882**	**0.869**	**0.943**	**0.888**
**2015**	**0.838**	**0.844**	**0.796**	**0.907**	**0.859**
**2016**	**0.765**	**0.768**	**0.726**	**0.848**	**0.788**
**2017**	**0.678**	**0.689**	**0.616**	**0.790**	**0.689**

*Adjusted odds ratios for antibiotic prescribing by specialty of facility and consultation year were determined from the multivariate logistic regression model including the following covariates: patient characteristics (sex, age group, and insurance status), facility characteristics (facility operation type and visiting hours), and seasonal fluctuation (month).

Abbreviations: ENT, ear, nose, and throat

## Discussion

This study provides important findings on the recent practice and trend of antibiotic prescribing for NB-ARTI in Japan. We found that the mean monthly antibiotic prescribing rate for NB-ARTI was 31.7% during the study period, which is lower than previously reported in Japan [9–11]. This difference is probably attributed to methods used to link analysed datasets. Our analyses were unique in that we examined the antibiotic prescribing rate by taking into consideration simultaneous diagnoses potentially requiring antibiotic treatment; thus, we were able to assess the appropriateness of antibiotic prescribing carefully. Our results were relatively similar to results from studies conducted in the US, which used rigorous conditions to assess the appropriateness of antibiotic prescribing and showed that the antibiotic prescribing rate for viral upper respiratory tract infections was 26% [4,14].

The proportion of broad-spectrum antibiotics prescribed among all antibiotics prescribed for NB-ARTI in the dataset was similar to that reported by a previous study that used the same data source [11]. According to the study using sales data, the proportion of broad-spectrum antibiotics (in DDD/1,000 inhabitants/day) among all prescribed oral antibiotics in Japan was about 76% in 2013 [3]. We found that broad-spectrum antibiotics comprised 90% of all antibiotics prescribed for NB-ARTI, suggesting that broad-spectrum antibiotics were more likely prescribed for NB-ARTI than other diagnoses. We obtained a similar prescribing rate for broad-spectrum antibiotics per NB-ARTI diagnosis as the previous study that used the same data source [11]. One of the reasons for the high proportion of broad-spectrum antibiotics prescribed in Japan might be clinicians' perception that broad -spectrum antibiotics are effective treatment for various common infections which led to their preference for broad-spectrum antibiotics [15].

Importantly, we found a trend or antibiotic prescribing for NB-ARTI to decline. However, the simple regression analysis of the trend in the NB-ARTI diagnosis rate per ARTI consultation versus the antibiotic prescribing rate per NB-ARTI consultation showed that this negative trend in prescribing rate was not associated with the downward trend in the number of diagnoses. Even with consideration of changes in the diagnostic rate, the decrease in antibiotic prescribing rate remains obvious. Based on multivariate analysis results, the antibiotic prescribing rate has significantly decreased since FY2012, especially in paediatrics clinics, even after controlling for confounding factors related to the antibiotic prescribing rate. In Japan, most local governments/municipalities subsidize children’s healthcare costs, and children can receive healthcare services free of charge in most cases [16]. Therefore, we initially assumed that this “local subsidy system” would promote consultation visits or antibiotic prescribing practices by physicians in paediatric clinics and paediatric population [17]. However, this study revealed contradictory results. Japanese paediatricians started ASP earlier than NAP was initiated, which probably contributed to the reduction of antibiotic use for NB-ARTI among paediatricians. In contrast, antibiotics tended to be prescribed more frequently for NB-ARTI at ENT (otolaryngology) clinics than at internal medicine or paediatric clinics. This finding suggests that there is a need to conduct education and awareness raising activities for physicians in select specialties.

In this study, patients of working-age who are employed and registered as members of the NHI were identified as factors associated with inappropriate antibiotic prescribing. A previous study conducted in Japan also showed that patients aged 20–29 years were likely to receive antibiotic prescriptions [11]. Other studies also reported that adults were likely inappropriately prescribed antibiotics [12,18]. Several studies reported that physicians tend to prescribe antibiotics in accordance with the patients’ expectations not only in Japan but also worldwide [19–23]. Many adults erroneously believe that antibiotics are effective against viral infections such as colds, influenza, and sore throat [24–26]. Therefore, it may be beneficial to promote education and awareness campaigns to the general public. In Europe, the internet is used to provide reliable health information that promotes appropriate antimicrobial practice [27,28]. However, In Japan it is not yet common to use the internet to provide health information; instead, people tend to receive information directly from health professionals [20,29]. However, since 2017, AMR Clinical Reference Centre in Japan has been disseminating information regarding antimicrobial stewardship through its website and social networking service (in Japanese) to the general public, which is expected to improve public knowledge.

We also found that antibiotics were prescribed more often for NB-ARTI in clinics with and without beds than in outpatient departments inside general hospitals. In particular, 90% of antibiotic prescriptions for NB-ARTI were issued by clinics without beds, which account for 85% of medical healthcare institutions in Japan [30]. Previous studies indicated that inappropriate antibiotic use was partly caused by prescriber factors such as clinicians’ poor knowledge on guidelines, clinicians’ later career stage, high daily patient volumes, and prior prescribing pattern or habit [23,31]. Clinics without beds in Japan are usually operated by a physician where peer pressure to check the appropriateness of prescriptions would be absent [32] and there would be no facility-wide ASP. Our study results suggested that these clinics are also important targets of ASP, including education and feedback.

The factors associated with antibiotic prescribing for ARTI have been studied in other countries. These factors include time pressure, the patient expectation for antibiotics, diagnostic uncertainty, and poor doctor-patient communication [8,21,22]. Due to the differences in study design and definition used, a simple comparison of the results of our study to those previous studies is difficult. However, identified factors associated with inappropriate antibiotic prescription in our study, such as patients of working-age, clinic types, and specialties of clinics may be associated with patient expectation for antibiotics, time pressure, and diagnostic uncertainty, respectively. As multiple previous studies focused more on prescribers’ factors[8,21,22], identifying local patients’ factors, such as patients of working-age identified in this study, seems also important to find appropriate targets of ASP.

This study has several limitations. Because JMDC is a database comprising data from large companies’ insurance union, data from retired employees are not included. We analysed the appropriateness of antibiotic prescribing based on the diagnosis obtained from medical claims submitted for reimbursement in the NIH system; therefore, diagnoses used in this study were not necessarily validated with actual clinical symptoms. Unfortunately, there is no robust information and there have been no studies to validate the diagnoses listed in medical claims in Japan. Based on the actual handling of medical claims in Japan, we processed the dataset by linking the consultation data file and the diagnosis data file under the condition of ≤ 30-day gap between the consultation date and the diagnosis date. Because the accuracy of the date of diagnosis is vague, there is a possibility that the antimicrobial prescribing rate is different according to the method used to link these datasets. In addition, there might have been minor discrepancies between the specialty listed in the JMDC database and the actual specialty at each facility,because JMDC extracted information regarding the specialty only the first listed specialty registered in the list kept by the Japanese Ministry of Health, Labour, and Welfare (i.e. if the facility has multiple specialties, specialties not listed in the first line would be missed).

In conclusion, our study revealed longitudinal trends and detailed characteristics of antibiotic prescribing for NB-ARTI in Japan. The results also provided insights into the inappropriate practice of antibiotic prescribing for NB-ARTI in Japan, which highlights optimal targets for ASP in outpatient settings. Education and awareness for adults and promotion of ASP among physicians in clinics without beds will be key drivers to reduce inappropriate antibiotic prescribing in Japan.

## Supporting information

S1 Fig Trend of monthly antibiotic prescribing by age group during the study period. Abbreviations: J01DD, third-generation cephalosporins; J01FA, macrolides; J01MA, fluoroquinolones; J01C, penicillins; Total Abx, total antibiotics prescribing for non-bacterial acute respiratory tract infections.
(DOCX)

S1 Table Appropriateness of antibiotic prescribing for ARTI used in the study. (DOCX)

S2 Table Sensitivity analysis of antibiotic prescribing rate by changes in linkage of the diagnoses dataset and the consultation dataset. (DOCX)
