# Supplementary material for: Longitudinal trends of and factors associated with inappropriate antibiotic prescribing for non-bacterial acute respiratory tract infection in Japan: A retrospective claims database study, 2012–2017
Source: PLoS One. 2019 Oct 16;14(10):e0223835. doi: 10.1371/journal.pone.0223835 (PMC6795458; doi:10.1371/journal.pone.0223835)
Supplement: S1 Fig — Abbreviations: J01DD, third-generation cephalosporins; J01FA, macrolides; J01MA, fluoroquinolones; J01C, penicillins; Total Abx, total antibiotics prescribing for non-bacterial acute respiratory tract infections. (DOCX) [file pone.0223835.s001.docx]

**S1 Figure.** Trend of monthly antibiotic prescribing by age group during the study period.

Abbreviations: J01DD, third-generation cephalosporins; J01FA, macrolides; J01MA, fluoroquinolones; J01C, penicillins; Total Abx, total antibiotics prescribing for non-bacterial acute respiratory tract infections
