# Supplementary material for: Longitudinal trends of and factors associated with inappropriate antibiotic prescribing for non-bacterial acute respiratory tract infection in Japan: A retrospective claims database study, 2012–2017
Source: PLoS One. 2019 Oct 16;14(10):e0223835. doi: 10.1371/journal.pone.0223835 (PMC6795458; doi:10.1371/journal.pone.0223835)
Supplement: S2 Table — (DOCX) [file pone.0223835.s003.docx]

**S2 Table** Sensitivity analysis of antibiotic prescribing rate by changes in linkage of the diagnoses dataset and the consultation dataset

| Number of days between the date diagnoses given and the consultation date (days) | Freq. of consultations which antibiotics prescribed (n) | Proportion of consultations which antibiotic prescribed for NB-ARTI(%) |
| --- | --- | --- |
| 0 | 4,446,064 | 36.34 |
| 30 | 5,383,557 | 31.28 |
| 60 | 5,452,973 | 30.72 |
| 90 | 5,470,177 | 30.54 |
| 120 | 5,477,387 | 30.45 |
| 150 | 5,481,265 | 30.4 |
| 180 | 5,483,950 | 30.36 |
| 210 | 5,485,885 | 30.33 |
| 240 | 5,487,362 | 30.3 |
| 270 | 5,488,500 | 30.29 |
